# Enhanced Antibacterial Properties of Titanium Surfaces through Diversified Ion Plating with Silver Atom Deposition

**DOI:** 10.3390/jfb15060164

**Published:** 2024-06-16

**Authors:** Everton Granemann Souza, Chiara das Dores do Nascimento, Cesar Aguzzoli, Elena Sarai Baena Santillán, Carlos Enrique Cuevas-Suárez, Patricia da Silva Nascente, Evandro Piva, Rafael Guerra Lund

**Affiliations:** 1 Graduate Program in Electronic and Computer Engineering, Catholic University of Pelotas, Pelotas 96015-560, Brazil; chiara.nascimento@ucpel.edu.br; 2 Graduate Program in Materials Science and Engineering, University of Caxias do Sul, Caxias 95070-560, Brazil; caguzzol@ucs.br; 3 Academic Area of Dentistry, Autonomous University of Hidalgo, Pachuca de Soto 42080, Mexico; elena_baena8622@uaeh.edu.mx (E.S.B.S.); cecuevas@uaeh.edu.mx (C.E.C.-S.); 4 Biology Institute, Federal University of Pelotas, Pelotas 96010-560, Brazil; patricia.nascente@ufpel.edu.br; 5 Department of Restorative Dentistry, School of Dentistry, Federal University of Pelotas, Pelotas 96010-610, Brazil; piva@ufpel.edu.br (E.P.); rglund@ufpel.edu.br (R.G.L.); 6 Graduate Program in Materials Science and Engineering, Technology Development Center, Federal University of Pelotas, Pelotas 96010-610, Brazil

**Keywords:** Diversified Ion Plating (DIP), biofilm prevention, silver ion implantation, *Staphylococcus aureus* inhibition

## Abstract

In this study, we investigate the antibacterial effect of silver atoms implanted into a thin surface layer of titanium at low energies using an alternative ion plating technology called Diversified Ion Plating. Silver atoms were incorporated into titanium samples using reactive low-voltage ion plating at 2 keV and 4 keV. Surface modifications and morphology were evaluated using wettability, profilometry measurements, and energy-dispersive spectroscopy. For a precise determination of the quantity and depth of implanted silver atoms on titanium surfaces, a combination of experimental techniques such as Rutherford Backscattering Spectrometry along with Monte Carlo simulations were utilized. To assess the antibacterial effects of the silver atoms incorporated into pure titanium surfaces, bacterial suspension immersion tests were performed with a standard strain of *Staphylococcus aureus* (ATCC 12600). The outcomes indicate that titanium surfaces implanted with silver atoms were more effective in inhibiting the growth of *Staphylococcus aureus* than pure titanium surfaces. Better results were found when the deposition was performed at 4 keV, indicating that a deeper implantation of silver, spanning a few nanometers, can result in a longer and more effective release of silver atoms. These findings suggest the potential for the development of new, cost-effective biomaterials, paving the way for improved implant materials in various health-related applications.

## 1. Introduction

A bacterial biofilm is a complex, organized community of bacterial cells that are surrounded by a self-generated polymeric matrix and firmly attached to both living and non-living surfaces [1,2]. The ability to attach to surfaces serves as a protective mechanism that enables organisms to survive in challenging environments. This attachment facilitates the capture of nutrients, as suspended organic material in liquids settles onto the surface [3].

Biofilms may impact human health beneficially or detrimentally; however, they are more often associated with pathogenic forms of human diseases [4]. The presence of biofilm on implanted medical devices, such as central venous catheters and artificial heart valves, is a major cause of bacteremia, a condition characterized by the presence of bacteria in the bloodstream, which, if not promptly treated, can lead to a range of serious health complications [5]. In the field of dentistry, the formation of biofilms on restorative materials is a common occurrence that frequently provokes the onset of dental caries [6,7].

*Staphylococcus aureus* (*S. aureus*) is a common cause of community and hospital-acquired bacteremia. The estimated incidence of *S. aureus* bacteremia (SAB) is between 20 and 30 cases per 100,000 persons per year [8]. It is also prevalent in the oral mucosa, especially among patients who wear dental prostheses [9]. It is one of the bacteria responsible for denture stomatitis and can release toxins [10,11,12]. It is highly resistant to antibiotics, a result of metabolic changes in biofilm cells, which also influence drug permeability [13,14].

There are many strategies to prevent the creation of biofilms, with the most common one being surface modification [15,16]. Some authors report a reduction in biofilm formation when using specific bindings of antimicrobial peptides to surfaces of titanium [17]. The use of noble metals nanoparticles [18,19,20,21,22], polymer coatings on material surfaces [23], and changing surface topography [24] are another successful techniques that reduce or eliminate bacterial growth. Although electroplating is a widely used technique for surface modifications [25], ion implantation stands out among the mentioned techniques due to its superior precision in controlling dose levels [26,27,28,29].

Depending on the ion/target interactions, a low-energy source will introduce ions into the near-subsurface regions, whereas a high-energy source will increase the dose at significantly greater depths [30].

Our research investigates an alternative technology to conventional ion implantation techniques, which typically employ high implantation energies of up to 100 keV. Our method, known as Diversified Ion Plating (DIP) [31], offers greater viability from an industrial standpoint due to its short processing time, low-energy regime, and capability for implantation in complex geometries using a rotating-planetary system. This process is readily scalable to industrial production, yielding high production rates with a relatively simple setup at low energy costs.

To illustrate the effectiveness of the method, silver atoms were incorporated into a thin surface layer of pure titanium using reactive low-voltage ion plating at two different, very low energies (2 keV and 4 keV), aiming to explore the effects of silver at two different depths. The quantity of implanted silver was determined through quantitative analysis using Rutherford Backscattering Spectroscopy (RBS) measurements and Monte Carlo simulations. Meanwhile, the analysis of surface modification was carried out by examining changes in wettability and roughness.

To assess the antibacterial efficacy of Ag^+^ after implantation, bacterial suspension immersion tests were conducted against biofilms of a collection strain of *Staphylococcus aureus* (ATCC 12600), which is commonly used for taxonomy purposes [32]. The results of these tests were compared with those obtained from pure titanium surfaces (control). The capacity of this uncomplicated procedure to implant silver atoms (as well as other metals) at relatively low energies offers new possibilities for the advancement of metallic biomaterials that possess antibacterial properties. This progress holds significant potential for boosting the safety and effectiveness of different medical devices, including orthopedic and dental implants, catheters, and other equipment used in medical procedures. These biomaterials can potentially lower the incidence of infections related to the devices, thereby enhancing the quality of life and health outcomes for patients.

## 2. Materials and Methods

### 2.1. Experimental Setup

#### 2.1.1. Substrates

To prepare the samples for implantation, unalloyed commercially pure titanium (CP Ti) grade 1, provided by Sandinox Biomateriais (Sorocaba, SP, Brazil), was used. The CP Ti grade 1 samples were cut into square pieces with dimensions of 10 × 10 × 0.5 mm. The chemical composition of the CP Ti grade 1 samples adhered to the specifications outlined in ASTM F67 [33], with the following composition: N: 0.03, C: 0.10, H: 0.0125, Fe: 0.20, O: 0.18, and balance Ti (wt%).

Before the implantation process, the samples were washed for 30 min in acetone using an ultrasonic cleaner and were then dried in a hot air stream to remove any residual contaminants. After the surface treatment was complete, the samples were stored in vacuum desiccators to maintain their purity until the implantation process.

#### 2.1.2. Diversified Ion Plating Process

**Figure 1:**
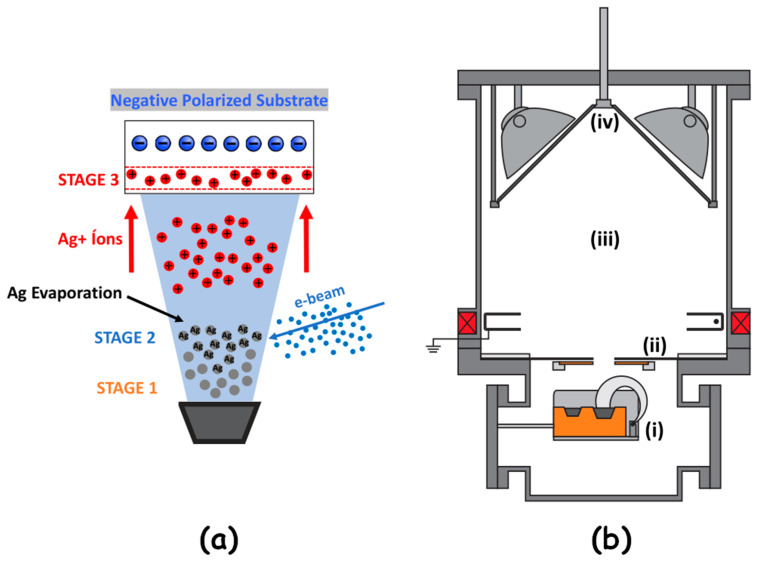
(**a**) Simplified schematic of the implantation process stages and (**b**) the main components of the ion plating equipment.

The DIP process was conducted using a reactive, low-voltage, ion plating-type device, following the methodology adopted by Echeverrigaray et al. [34,35]. During the ion implantation process, the following three stages occur, as shown in Figure 1a:

Stage 1—Electron Emission and Material Sublimation: In the first stage, a high current and voltage are applied to the electron gun’s source, heating the tungsten-thorium filament (cathode) and causing electron emission through the thermionic effect. A magnetic field directs these electrons to the center of the crucible, resulting in the sublimation of the silver pellets.

Stage 2—Ionization of Evaporated Metal Atoms: In the second stage, an electron beam (e-beam) is generated to ionize the evaporated silver atoms, converting them into positively charged ions as they travel through the vacuum chamber.

Stage 3—Ion Acceleration and Substrate Penetration: In the third stage, the produced ions are accelerated towards the polarized substrate, which has a negative potential (−2 kV or −4 kV for this study), using electrostatic attraction. Upon penetrating the substrate, the ions collide with the target titanium atoms, dissipating kinetic energy and becoming neutralized within the material’s crystal lattice.

From an equipment perspective, Figure 1b illustrates the main components, which include (i) the electron gun, (ii) the iris diaphragm, (iii) the implantation chamber, and (iv) the sample holder, which is biased with high voltage (BIAS) and serves as the holder for titanium substrate.

Regarding the process parameters, the vacuum pressure during implantation was maintained at 5.0 × 10^−5^ Pa, while the base pressure was kept at 1.0 × 10^−5^ Pa. Silver pellets with a purity of 99.9% used in the process were provided by Kurt J. Lesker Company (Jefferson Hills, PA, USA). The reactor was purged with nitrogen gas obtained commercially to remove any residual gases or contaminants. The operational parameters for the ion implantation process are specified as follows: the voltage applied to the electron source is set to 6.0 kV, the emission current is maintained at 25.0 mA, the filament current is fixed at 15.0 A, and the energy bias is adjusted to 2.0 and 4.0 keV. Consistent doses of silver were achieved under these process conditions. The samples were fabricated in 2022 and were utilized for a period of six months after production.

It is worth noting that the duration of ion implantation has a direct impact on the concentration of silver ions in the samples. An increase in process time and acceleration energy of ions is positively correlated with higher concentrations. For the purpose of our investigation, a 60 min implantation time was utilized for both implantation energies.

#### 2.1.3. Stoichiometry 

To determine the composition and concentration of silver atoms/cm^2^ implanted in CP Ti samples, Rutherford Backscattering Spectrometry (RBS) was utilized. The RBS measurements were obtained by impinging a 2 MeV He^+^ ions perpendicular to the sample surface at a scattered angle of 165°, similar to the method employed by [28,36]. The samples were attached to a grounded sample holder using metal tabs, and typical measurement times of 10–30 min were used for each sample. The accuracy of the energy peaks was determined by a calibration curve that included measurements from heavy elements to convert channel numbers to corresponding energy values.

#### 2.1.4. Surface Morphology and Elemental Composition

Scanning electron microscopy (SEM) was employed to evaluate the morphology of 1.0 × 1.0 × 0.2 cm-sized samples using the TESCAN VEGA3 model, operating at 20 kV with a magnification of 150×. The elemental composition of the samples was appraised through energy-dispersive spectroscopy (EDS), which was integrated with SEM using the Bruker Nano XFlash Detector 6–10 for chemical mapping.

#### 2.1.5. Wettability

In order to assess the wettability characteristics of the samples, the sessile drop technique was employed using a goniometer Model 300, manufactured by SEO Phoenix, Seoul, Republic of Korea. The technique involved generating three micrometer-scale droplets of distilled water for each sample and subsequently measuring each droplet at ten distinct locations. The contact angle was determined using the image analysis program, Surftens 3.0.

#### 2.1.6. Surface Roughness

Surface roughness was measured using a Taylor Hobson device, model 112/2009, with a tip radius of 2 µm and a cone angle of 90°. To ensure the reliability of the results, the average roughness profile (*R_a_*) was determined for three distinct regions of the samples, and the average and standard deviation were calculated to express the results.

#### 2.1.7. Bacterial Suspension Immersion Tests

The antibacterial activity was evaluated against the standard strain of *Staphylococcus aureus* (ATCC 12600). To prepare the bacterial inoculum, the strain was cultivated in Brain Heart Infusion (BHI) broth (Difco, Sparks, MD, USA) at 37 °C for 24 h until it reached the exponential growth phase. Subsequently, the cultures were adjusted via dilution to a turbidity equivalent to 0.5 McFarland scale (approximately 1.5 × 10^8^ CFU/mL).

The biofilm formation was quantified using the conventional titer plate method with slight modifications following a comparable protocol to that outlined by Peralta et al. [37]. To evaluate biofilm formation, a bacterial inoculum was prepared by diluting 1:100 in fresh BHI (total 1.980 mL) and cultured in a 24-well microtiter plate containing nine titanium samples of 1 cm^2^ (sextuplicates of CP Ti grade 1 (Ti), CP Ti grade 1 implanted with Ag at 2 keV (Ag/Ti2), and CP Ti grade 1 implanted with Ag at 4 keV (Ag/Ti4)). The monoculture samples were incubated at 37 °C for 72 h after inoculation to induce biofilm formation.

At 24 h intervals, the specimens underwent a cleansing process utilizing a 0.9% NaCl (saline) solution, followed by complete replacement of the culture medium, resulting in the retention of solely the sessile cells within the medium.

After 72 h, the specimens were retrieved and rinsed using 0.9% NaCl solution to eliminate detached cells. The biofilm-containing specimens were then transferred to a 1 mL Eppendorf tube filled with a 0.9% NaCl solution and sonicated for 30 s at 30 W using an S500 Sonicator (R2D091109, Brazil). This process resulted in the complete resuspension of the biofilm in a sterile saline solution.

Subsequently, the suspensions underwent serial dilutions until reaching a maximum inoculum dilution equivalent to 10^−7^. Then, two 10 µL aliquots from each Eppendorf were plated on BHI agar. The resulting samples were then incubated at 37 °C for 24 h to quantify the number of colonies forming units (CFUs). Thus, the CFUs were calculated in CFU/cm^2^ and expressed on a logarithmic scale (log_10_).

#### 2.1.8. Statistical Analysis

The results displayed for the microbiological assays were conducted for an average of six replicates per sample type, namely Ti, Ag/Ti2, and Ag/Ti4, considering a dilution factor of 10^−4^, which provided the highest colony count within the range of 300 to 30 CFU.

The consistency of the dataset was assessed using Grubb’s test to detect the presence of any outliers, which were then removed. Normality and homogeneity of variance were checked for the remaining data. To evaluate the impact of the dependent variable on the CFU/cm^2^, a one-way analysis of variance was performed, followed by Bonferroni’s post hoc test. The significance level was set at α = 5%. The null hypothesis tested was that the modification of titanium surfaces with silver atoms would not enhance the antibacterial effect against Staphylococcus aureus. Additionally, for contact angles and arithmetic surface roughness (*R_a_*), the null hypothesis tested was that the modification of titanium surfaces with silver atoms would not significantly influence surface roughness or wettability.

## 3. Results and Discussion

### 3.1. Surface and Chemical Analysis

DIP is a reactive low-voltage implantation process, here represented by the implantation of Ag^+^ ions into titanium samples at low energies of 2 keV and 4 keV. Although highly controllable, the main challenge in the DIP process for industrial/medical purposes is to precisely implant a specific amount of silver atoms into a titanium surface. This process requires a delicate balance in the amount of silver for efficient bactericidal action.

Adhesion followed by bacterial inhibition/eradication is a complex and multifactorial process [38] that involves both surface properties of materials as well as bacterial and microbial environmental characteristics. Factors such as roughness, wettability, and chemical composition of the surface are important to prevent bacterial adhesion. Concentration, release kinetics of ions, exposure time, bacterial cell wall characteristics, motility, and bacterial density are critical parameters for the survival of the microorganisms [39].

**Figure 2:**
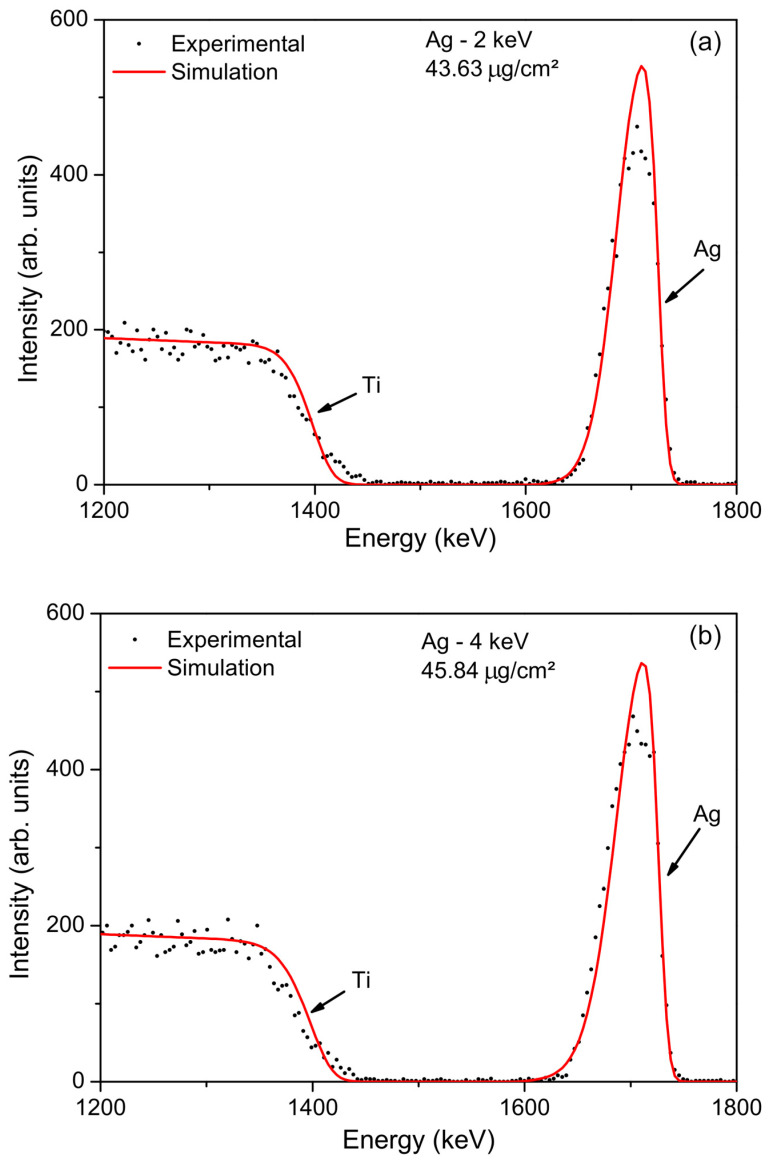
RBS spectrum of the Ag/Ti samples for implantation energies of (**a**) 2 keV and (**b**) 4 keV. The experimental data are represented by discrete black dots, while the simulations with software SINMRA version 7.03 [41] are denoted by a continuous red line. The arrows indicate the surface energy of each element.

RBS is a non-destructive technique that offers high precision and accuracy for quantifying the amount of silver present in the samples. This technique is particularly sensitive to variations in atomic number and can achieve high depth resolution [40]. Figure 2 shows the RBS spectrum with characteristic signals of both titanium (substrate) and the incorporated Ag atoms.

The presence of the silver signal in the spectrum confirms the successful implantation of Ag^+^ ions into the titanium surface. Due to the low implantation energy and the greater atomic mass of silver compared to titanium, the back-scattering peak associated with silver is more pronounced than the corresponding peak for titanium. The oxygen peak, present in the native Ti layer, is not detected due to its low atomic number, combined with the small thickness of the native oxide layer.

Besides identifying the sample’s constituents, no characteristic peak of any other chemical element was detected. Therefore, it is possible to infer that the sample does not contain any contaminants, or, if present, their quantity is below the resolution limit of the technique.

Based on the RBS measurements, the total amount of silver atoms present in the CP Ti substrate was similar and determined to be 2.43 × 10^17^ at/cm^2^ (43.63 µg/cm^2^) for 2 keV and 2.55 × 10^17^ at/cm^2^ (45.84 µg/cm^2^) for 4 keV.

**Figure 3:**
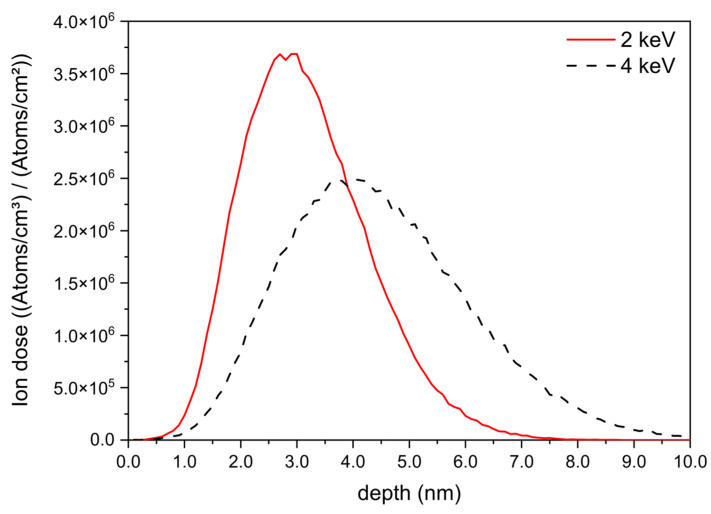
Simulation showing the in-depth silver concentration profiles inside titanium for implantation energies of 2 keV and 4 keV.

Figure 3 depicts a comparative analysis of simulated profiles, using the SRIM software package version 2013 [42], of equivalent silver ion implantation quantities in titanium with energy levels of 2.0 keV and 4.0 keV. The maximum concentration of Ag^+^ ions at 2.0 keV penetrates to a depth of approximately 3 nm, with a subsequent spreading, following a gaussian shape, to a maximum depth of 7 nm. In contrast, for 4.0 keV energy, the maximum concentration of Ag^+^ is achieved at 4.0 nm, where Ag is implanted slightly deeper and distributed over a broader depth of 10 nm.

**Figure 4:**
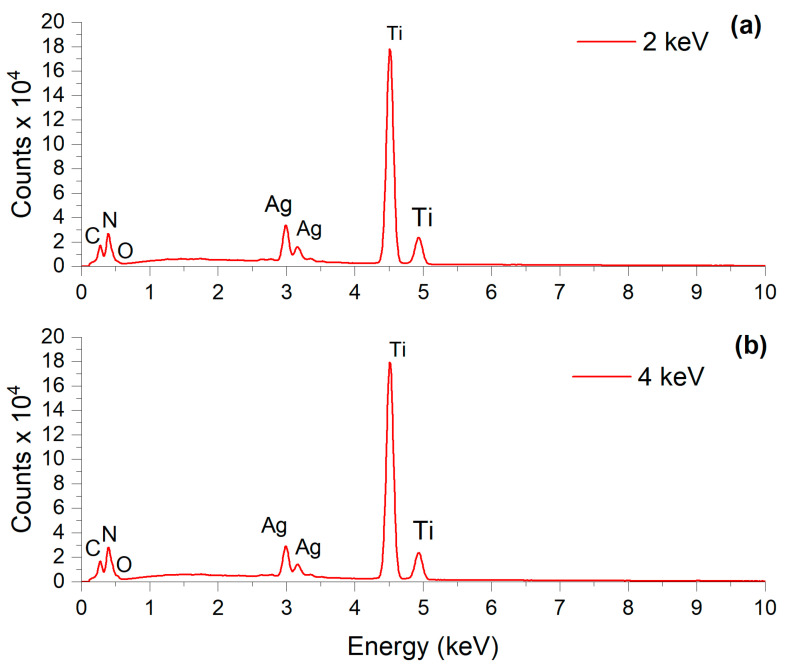
EDS spectrum of titanium samples with silver implantation at (**a**) 2 keV and (**b**) 4 keV.

In order to complement the surface analysis provided by RBS, energy-dispersive X-ray spectroscopy (EDS) was used to map the elemental composition across the surface of the films. The results are shown in Figure 4.

As evident, for both implantation energies, the prominent titanium peak indicates its abundance as the substrate element. The minor silver peaks suggest the presence of silver, albeit in lesser quantities compared to titanium. Peaks of carbon, nitrogen, and oxygen imply additional elements, likely attributable to surface contaminants or substrate components. 

The reason the Ag signal in Figure 4a (2 keV) is slightly higher than in Figure 4b (4 keV) lies in the EDS signal’s generation. In the 2 keV case, the Ag atoms are closer to the sample surface, leading to a greater absorption of X-rays emitted by the Ti atoms beneath them. Therefore, the current signal ratio is not due to concentration but rather due to the complex absorption/fluorescence sequence and the spatial distribution of the respective elements.

Although EDS provides a qualitative measure, it corroborates the RBS result, indicating the presence of silver for both implantation energies. 

The wettability of the surface also influences the interaction between the liquid medium, the bacterial inoculum, and the modified titanium surfaces. When a liquid comes into contact with a solid surface, it wets the surface by spreading out to increase its contact area. This phenomenon is determined by the contact angle, which is the angle formed by the liquid–solid interface at the point of contact. A low contact angle indicates high wettability, meaning that the liquid spreads out over the surface, while a high contact angle indicates low wettability, meaning that the liquid beads up on the surface. The second column of Table 1 presents the contact angle values for pure titanium (Ti) and titanium implanted with silver at 2 keV (Ag/Ti2) and 4 keV (Ag/Ti4).

The contact angle measurements of Ag/Ti2 and Ag/Ti4 were found to be lower than that of the pure substrate, Ti, indicating that the surface of titanium becomes less hydrophobic after silver is implanted [43]. Normally, bacteria tend to preferentially adhere to hydrophobic surfaces, which exhibit higher contact angles [44]; however, we believe that such a decrease in the contact angle between Ti, Ag/Ti2, and Ag/Ti4 would not be sufficient to significantly reduce the formation of biofilm. 

The implantation of the silver atoms could also change the surface roughness. The presence of surface irregularities creates microenvironments that provide favorable conditions for the retention of nutrients and water, enabling bacterial proliferation and biofilm formation.

The third column of Table 1 displays the surface roughness values *R_a_* obtained for Ti, Ag/Ti2, and Ag/Ti4, which represent the arithmetic mean of the absolute values of the surface irregularities’ heights with respect to a mean line.

It can be observed that all the *R_a_* values are near to or below 0.20 µm, indicating that all the surfaces are relatively smooth and uniform, with few small irregularities. Although the existence of a threshold roughness is currently debated, many studies have shown that surfaces with *R_a_* roughness values below 0.2 µm tend to be less prone to biofilm formation [45].

Among the samples analyzed, Ag/Ti2 exhibits the lowest *R_a_* value, which may explain its greater hydrophilicity (97.3° ± 0.48) displayed in Table 1. In general, smoother surfaces tend to exhibit lower contact angles and better wettability.

### 3.2. Statistical Results of the Microbiological Essay

The strong antimicrobial effects of Ag ions and Ag-based compounds are widely recognized [46]. The discovery of antibiotics in the early 20th century caused a decline in the use of silver as an antimicrobial agent. Nevertheless, the growing levels of bacterial resistance to many antibiotics in recent years have prompted a re-evaluation of the potential of this age-old remedy [47].

There are several ways to use silver as an antimicrobial agent. Some of the most common forms include salts, nanoparticles, colloids, and ions. While all these forms have their benefits, in general, Ag^+^ ions have certain advantages over silver nanoparticles (AgNPs), colloids, and salts, especially in terms of safety [48].

**Figure 5:**
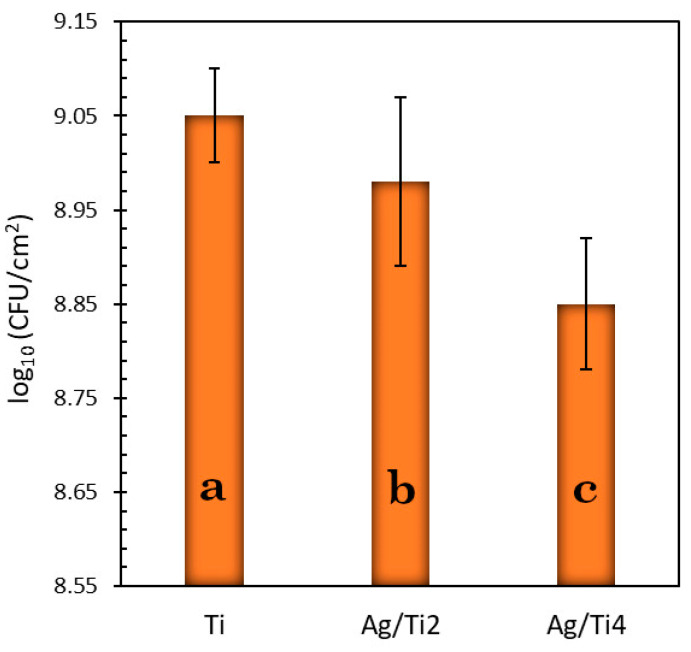
CFU/cm^2^ of *S. aureus*, in log_10_ scale, for pure (control) and silver-implanted samples of titanium at energies of 2 and 4 keV. Different lowercase letters indicate the presence of statistically significant differences (*p* < 0.05).

The bioactivity of Ag^+^ implanted on a Ti surface was tested against the Gram-positive bacteria *S. aureus* (ATCC 12600) using bacterial suspension immersion tests. Titanium samples were exposed directly to a suspension of bacteria (as detailed in the Bacterial Suspension Immersion Tests section) and were then incubated under controlled conditions to allow the formation of a biofilm. The quantification of colony-forming units per square centimeter (CFU/cm^2^) was determined for each sample, and the averaged values were derived from six replicates. The obtained results, presented in Figure 5, are presented on a logarithmic scale.

By observing the results, it is evident that the pure Ti (control) sample showed the highest bacterial growth since it did not contain any silver. Although the Ag/Ti2 sample had a slightly lower contact angle and roughness, which could potentially provide some advantages in terms of limiting colony growth, it did not demonstrate superior antimicrobial action compared to the Ag/Ti4 sample. This suggests that the presence of Ag^+^, at the concentration (45.84 µg/cm^2^) and depth (10 nm) used in this study, is a dominant factor in inhibiting bacterial growth, and it overrides the differences in roughness and wettability observed in the Ag/Ti2 sample (Table 1).

This depth causes the Ag^+^ silver ions to be distributed in a thicker layer on the sample surface, which leads to a larger contact area between the ions and bacteria, which constitutes to the presence of a negative electrical charge on its cell surface. This, in turn, may result in a slower, more gradual release of silver ions into the bacterial suspension, potentially increasing the effectiveness of silver as an antimicrobial agent. Similar behavior was found by Mohamed et al. when they used silver-killed *E. coli* O104:H4 to kill *E. coli* O157:H7, multidrug-resistant (MDR) Pseudomonas aeruginosa, and methicillin-resistant Staphylococcus aureus (MRSA) [49].

While silver demonstrates potent antimicrobial properties, prolonged exposure to high concentrations can pose a risk of toxicity to human cells. In previous investigations conducted by the research group, a comparable amount of silver was evaluated for its cytotoxic effects on human cells (MG-63), and no evidence of toxicity was found. Over a span of 1, 5, and 7 days, the presence of the extract from the treated sample did not result in any decrease in cell viability when compared to the negative control, which represents the optimal cellular condition [29]. These findings suggest that the tested concentration of silver extract does not adversely affect cell viability under the evaluated experimental conditions.

## 4. Conclusions

The present study investigated the antibacterial effects of silver atoms implanted into titanium surfaces using an alternative ion plating technology known as Diversified Ion Plating. The outcomes revealed that the DIP technique successfully incorporated silver atoms into pure titanium surfaces at effective energy levels of 2 keV (Ag/Ti2) and 4 keV (Ag/Ti4). The resulting surfaces exhibited notable inhibitory effects against *Staphylococcus aureus* (ATCC 12600) biofilms, surpassing the performance of commercially available pure titanium surfaces.

Although the Ag/Ti2 sample exhibited a slightly lower contact angle and roughness, which could theoretically offer some advantages in limiting colony growth, it did not demonstrate superior antimicrobial action compared to the Ag/Ti4 sample. This suggests that the presence of silver in a deeper layer ranging from 1 to 10 nm is the dominant factor in inhibiting bacterial growth, improving the antibacterial effects of silver atoms due to the locally relatively large Ag^+^ concentration in the near-surface fluid layer. This effect overrides the differences in roughness and wettability observed in the Ag/Ti2 sample, which has a narrower implantation depth range of 1 to 6 nm.

The findings of this study suggest the potential for the development of new, cost-effective biomaterials that incorporate silver atoms using the DIP technique, paving the way for the development of improved implant materials that may be used in a variety of health-related applications. Further studies are needed to evaluate the long-term stability and biocompatibility of these materials, and to explore their potential clinical applications.

## Figures and Tables

**Table 1:** Contact angles and the arithmetic surface roughness, *R_a_*, for pure titanium samples and samples implanted with silver energies of 2 keV and 4 keV. The quantity in parenthesis represents the standard deviation for an average of ten measurements. The different superscript letters indicate the presence of statistically significant differences (*p* < 0.05).

Sample	Contact Angle (°)	Contact Angle Image	*R_a_* (µm)
Ti	107.6 (1.01) ^a^	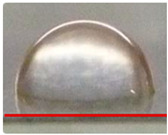	0.19 (0.01) ^a^
Ag/Ti2	97.3 (0.48) ^c^	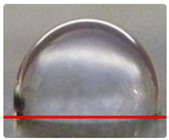	0.10 (0.03) ^b^
Ag/Ti4	102.7 (0.55) ^b^	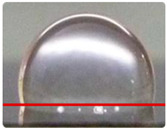	0.20 (0.01) ^a^

## Data Availability

The original contributions presented in this study are included in this article; further inquiries can be directed to the corresponding authors.
